# Dissemination of novel biostatistics methods: Impact of programming code availability and other characteristics on article citations

**DOI:** 10.1371/journal.pone.0201590

**Published:** 2018-08-01

**Authors:** Amy E. Wahlquist, Lutfiyya N. Muhammad, Teri Lynn Herbert, Viswanathan Ramakrishnan, Paul J. Nietert

**Affiliations:** 1 Department of Public Health Sciences, Medical University of South Carolina, Charleston, SC, United States of America; 2 Department of Library Science and Informatics, Medical University of South Carolina, Charleston, SC, United States of America; Public Health Agency of Canada, CANADA

## Abstract

**Background:**

As statisticians develop new methodological approaches, there are many factors that influence whether others will utilize their work. This paper is a bibliometric study that identifies and quantifies associations between characteristics of new biostatistics methods and their citation counts. Of primary interest was the association between numbers of citations and whether software code was available to the reader.

**Methods:**

Statistics journal articles published in 2010 from 35 statistical journals were reviewed by two biostatisticians. Generalized linear mixed models were used to determine which characteristics (author, article, and journal) were independently associated with citation counts (as of April 1, 2017) in other peer-reviewed articles.

**Results:**

Of 722 articles reviewed, 428 were classified as new biostatistics methods. In a multivariable model, for articles that were not freely accessible on the journal’s website, having code available appeared to offer no boost to the number of citations (adjusted rate ratio = 0.96, 95% CI = 0.74 to 1.24, p = 0.74); however, for articles that were freely accessible on the journal’s website, having code available was associated with a 2-fold increase in the number of citations (adjusted rate ratio = 2.01, 95% CI = 1.30 to 3.10, p = 0.002). Higher citation rates were also associated with higher numbers of references, longer articles, SCImago Journal Rank indicator (SJR), and total numbers of publications among authors, with the strongest impact on citation rates coming from SJR (rate ratio = 1.21 for a 1-unit increase in SJR; 95% CI = 1.11 to 1.32).

**Conclusion:**

These analyses shed new insight into factors associated with citation rates of articles on new biostatistical methods. Making computer code available to readers is a goal worth striving for that may enhance biostatistics knowledge translation.

## Introduction

Knowledge translation is fundamental to advancing science. There are multiple routes by which scientists disseminate their findings to aid in translation, but publishing findings in peer-reviewed journals is perhaps one of the most important means for doing so. For biostatisticians, as with most scientists who work in academic settings, publishing manuscripts that highlight contributions to their respective fields is key to career advancement, including promotion and tenure. Although publishing is personally important to the authors, it is important to recognize that reasons for publishing also include advancing the field of biostatistics and influencing the application of biostatistics methods to real-world settings [1–3].

Prior research suggests that the uptake of new statistical methods has much room for improvement [4]. Pullenayegum et al. (2016) provide some examples, including researchers not incorporating measurement error in regression models when indicated and the failure to utilize more adaptive designs in clinical trials [5]. Inverse-intensity weighting, one specific example of a class of statistical techniques designed in the early 2000’s to handle longitudinal data with random follow-up times related to the outcome [6], has rarely been implemented, despite this phenomenon occurring often in certain types of chart review studies. Prior to Pullenayegum et al.’s 2016 paper, the method had only been used once as a primary analysis [7]. A survey conducted by Canada’s Natural Sciences and Engineering Research Council also suggests that researchers in certain fields related to mathematics, including statisticians, engage in knowledge transfer less often than colleagues in other natural sciences and engineering disciplines [8]. In our own prior work, we found that only 1.7% of articles published in the field of general/internal medicine research from 2000–2009 included a citation of an article published in the biostatistics literature during that same time frame, perhaps providing further evidence of suboptimal translation of the knowledge gained through biostatisticians’ primary research [9].

While there are likely many ways that biostatisticians could improve the rate at which knowledge translation occurs, information within biostatistics publications themselves might provide insight into means of “successful” knowledge translation. Since biostatistics methods are algorithms, providing the algorithms in user-friendly formats to the reader, via printed computer code or some other means (e.g. on a personal or journal website, through e-mail upon request), could likely facilitate the use of that method by other scientists as well as help ensure reproducibility [10–13]. The ability to reproduce results is becoming more important as researchers’ analytic techniques and data structures become more complex while searching for weaker associations among variables. As researchers attempt to reproduce other investigators’ results and identify potential errors, having as much information as possible in primary publications (including accompanying data and/or code) will help ensure the reliability of the dissemination of new methods [11].

Researchers have promoted strategies for increasing citation frequencies, including publishing in journals that are read by large numbers of readers or that have high impact value, having the article freely accessible online (e.g. in a self-archive, public repository, or open-access format), including authors from multiple institutions and/or multiple countries, using more references, writing longer articles, sharing research data, and publishing across disciplines [14–16]. Publishing articles with tables and figures may also tend to increase readership as well [17], although this is practically universally done in biostatistics methods papers. Based on earlier findings suggesting that expository articles may tend to disseminate faster than traditional statistical methodological articles [18], we hypothesized that illustrating the use of the new method on a “real-world” dataset might also be associated with higher citation counts. We also recognize, however, the possibility that this might have the reverse effect if the examples are not used or presented appropriately.

This paper is a bibliometric study that illustrates the associations between characteristics of published articles summarizing new biostatistics methods and one knowledge translation metric, their citation counts. While all of these characteristics are of interest with regard to their relationship with citation counts, of primary interest was the extent to which making computer software code available to the reader is associated with future citations.

## Methods

To review biostatistical methods summarized in articles from a broad range of journals, we initially identified 85 peer-reviewed English-language journals that were listed among journals in the “Statistics and Probability” category for the year 2010, according to SCImago Journal & Country Rank[19]. From this list, we excluded journals that were not primarily focused on statistical methods or deemed not relevant to biostatistics, leaving 35 journals. Additionally, in Feb 2016, we conducted an informal 1-question e-mail survey of 49 biostatisticians linked to biostatistics, epidemiology, and research design (BERD) programs at institutions with Clinical and Translational Science Awards (CTSAs) that asked: “If you were going to submit a manuscript for publication that discussed a new biostatistical method (or an extension of an existing method) that you developed, in what journal would you prefer to publish? Please name up to 5 journals.” Among 20 responders (41% response rate), all but two biostatistics journals mentioned were already included in our list of 35; these two were added to our list of journals. We then randomly selected from each of these journals up to 20 articles whose publication date was in the calendar year 2010. For journals with fewer than 20 articles published in 2010, all articles were selected. The year 2010 was chosen to provide a reasonable amount of time (i.e. 7 years) for knowledge translation to have occurred and also to ensure that the articles in question reflect methods which are still relatively novel. The goal was to identify at least 400 articles considered to be new statistical methods published across a range of journals, in order to provide 80% power to detect modest differences (effect sizes equivalent to 0.4 standard deviation units in a 2-sample t-test framework) in citation counts, and modest increases in the proportion above the median numbers of citations (e.g. 45% vs. 62%, equivalent to an odds ratio around 2.0, in a 2x2 chi-square test framework), assuming 2-sided testing and an alpha level of 0.05.

An article was considered to be about a new biostatistical method if it described a novel statistical technique or algorithm or extended an existing technique or algorithm, which could potentially be used during the design, conduct, or analysis of a biomedical research study. If a method was statistical in nature but designed for a non-biomedical application (e.g. economics, agriculture), we did not necessarily exclude that article, since many statistical methods designed for non-biomedical disciplines are eventually adapted for use in biomedical research. Articles that compared already-existing biostatistical methods but did not extend those prior methods in any novel way were excluded, as were papers merely descriptive in nature about a particular study’s design, analysis, or results. Articles that appeared to be purely mathematical and/or statistical proofs were excluded, because in such cases the authors were not typically describing new techniques and/or algorithms that could be readily adapted for use in other studies. In using this type of rubric for classification, the intent was to be highly specific, meaning that there would be little doubt that the final list of journal articles all represented novel biostatistical methods. Questions that helped decide whether an article was outlining a new biostatistical method included: 1) Does the article describe something that could be used for helping design a study? 2) Does the article describe something that could be used for helping analyze data from a study? 3) Does the article describe something for which computer code could be used to help make use of this new method?

For articles classified as new methods, a number of characteristics were abstracted and entered into a REDCap database. These characteristics included publication dates (in print and on-line, if available), page counts (range: 1 to 54), number of references cited (range: 2 to 95), number of authors (range: 1 to 7), number of publications by most published author (range: 2 to 1653), total number of author publications (range: 3 to 1745), whether any of the authors’ institutional involvement was located in the U.S., whether the authors collectively were affiliated with more than one institution and/or country, whether there was evidence that any of the authors’ primary discipline was clinical or otherwise non-methodological (i.e. as indicated by their degree or affiliation), whether computer code was made available to the reader (i.e. in the actual article, appendix, or supplementary material; on a referenced web page; or upon request from one of the authors), and whether a real-life application of the method was provided in the article. If an article specifically referenced computer code uploaded or published elsewhere (e.g. CRAN, GitHub), then that article was classified as having provided computer code. The publication dates were used to create a representation of the duration of follow-up time, defined as the number of months from publication (on-line or in print, whichever was earlier) until April 1, 2017. We also recorded the article’s 2010 SCImago Journal Rank (SJR) indicator (range: 0.670 to 6.036), its journal’s h-index (range: 14 to 133), whether the article was available in the PubMed Central repository [19], whether the article was freely accessible on the journal’s website, and whether a version (e.g. pre- or post-print) of the article was published on any freely accessible website (including the journal’s website). For this study, both the SJR indicator and journal h-index provided journal ranking scores based on citations received from other publications. While the SJR indicator and h-index are both metrics that quantify citation rates, a journal’s SJR indicator is a broad measure of its citations over a 3-year time period, while a journal’s h-index is more reflective of its most highly cited articles. The full details for how these scores are calculated can be found on the SCImago Journal & Country Rank website [19]. Finally, we noted the number of citations each article had (as of April 1, 2017) and the number of publications each author had (as of May 1, 2017), according to the Scopus® citation database [19, 20]. Although it is possible that articles with higher citation counts may be of higher quality than others (i.e. more effective and reproducible), we did not specifically examine or judge the quality of the new methods being proposed in these articles.

Articles were reviewed and characterized by two of the three biostatisticians listed as authors (AEW and PJN, or LNM and PJN). When there was disagreement, for example, on whether an article truly represented a novel statistical method or whether code was available, the biostatisticians re-evaluated their assessments until consensus was reached. The biostatisticians agreed approximately 80% of the time on whether an article should be classified as a new method, but after discussion consensus was achieved 100% of the time. A complete list of the articles included in the final analyses and their abstracted characteristics is included as supporting information (S1 Dataset). The SAS code reflecting the analyses has also been submitted as supporting information (S1 File).

Since the primary hypothesis centered around whether making code available in the article would be associated with higher numbers of citations, characteristics of new methods articles with and without code available were compared using generalized linear mixed models (GLMMs) that included random journal effects to account for within-journal correlation. The various article characteristics served as dependent variables, incorporating an appropriate distributional assumption (e.g. binomial, multinomial, Poisson, or normal) and link function (e.g. log, linear), depending on whether the characteristic was a categorical, count, or continuous variable. A binary variable indicating whether the article had code available served as the independent variable in each of the models. A similar strategy was then used to determine which article, author, and journal characteristics were associated with the number of citations during the study follow-up period. For these analyses, we used a series of GLMMs to examine whether article characteristics (independent variables) were associated with number of citations (dependent variable). The models assumed a lognormal distribution for the number of citations, and each utilized a log link function. All of these GLMMs incorporated random journal effects to account for within-journal correlation. For each variable, a rate ratio was calculated by exponentiating the estimated regression coefficient; the rate ratio and its 95% confidence interval were reported. The rate ratio reflects the fold-increase in number of citations associated with having vs. not having the factor of interest (for categorical variables) or with a 1-unit increase (for SJR indicator and number of authors) or a 10-unit increase (for other continuous and count variables). Spearman correlations assessing the magnitude of the associations between number of citations and continuous characteristics were also calculated.

Finally, we created a multivariable GLMM using the number of citations as the dependent variable. Assuming a lognormal distribution for the number of citations provided superior model fit with normally distributed residuals, as assessed via diagnostic plots, when compared to models that assumed other distributions, including normal, Poisson, and negative binomial. In this process, all main effects for the characteristics were included. We also investigated all two-way interactions between an indicator variable representing whether or not code was available in the article and each of the remaining characteristics. Using a forwards selection approach, only a single significant interaction was added. When characteristics were moderately to highly correlated with each other (rho>0.5), only the one most correlated with number of citations was selected to be included in the multivariable model. The model included random journal effects to account for clustering of articles within journals. Since the lognormal modelling approach requires outcomes to be nonzero, n = 19 articles with 0 citations were not included in the primary multivariable model; however, we conducted a sensitivity analysis to see whether any bias was introduced by this omission by assessing whether the study conclusions changed when the natural logarithm of the number of citations plus 1 was treated as the dependent variable in the multivariable model. We also conducted a sensitivity analysis in which several articles with extremely high number of citations were excluded from the multivariable analyses.

## Results

A total of 722 articles were reviewed, of which 428 (59%) were classified as being novel biostatistics methods. Among the 428 new methods articles, 19 (4.4%) were never cited during the study follow-up period, and the maximum number of citations for an article was 535. The mean (± SD) number of citations was 16.7 (±38.9), and the median (interquartile range) was 8.0 (3–15). Table 1 lists descriptive statistics associated with the primary abstracted data elements, stratified by whether or not computer code was available. Characteristics were generally similar between articles with and without code available; however, compared to articles without code available, articles with code available were more likely to include a coauthor who was not a methodologist (9.6% vs. 3.8%, p = 0.04).

**Table 1:** Comparisons between studies with and without code available.

	**Code Available**				**Code Not Available**				
**Categorical variables**	**(n = 135)**				**(n = 293)**				P-Value*
At least 1 author is from a US Institution (n, % yes)	71, 52.6%				140, 47.8%				0.88
Multiple countries represented by authors (n, % yes)	31, 23.0%				80, 27.3%				0.34
Multiple institutions represented by authors (n, % yes)	80, 59.3%				173, 59.0%				0.95
Evidence that at least 1 author is not a methodologist (n, % yes)	13, 9.6%				11, 3.8%				0.04
Types of examples presented in paper									
No example provided (n, % yes)	1, 0.7%				17, 5.8%				0.67
Simulation only (n, % yes)	23, 17.0%				58, 19.8%				
Real-life example (n, % yes)	111, 82.2%				218, 74.4%				
Manuscript deposited into PubMed Central (n, % yes)	29, 21.5%				40, 13.7%				0.18
Manuscript freely accessible on journal website (n, % yes)	31, 23.0%				90, 30.7%				0.48
Manuscript freely accessible on any website (n, % yes)	87, 64.4%				185, 63.1%				0.22
	**Code Available** **(n = 135)**				**Code Not Available** **(n = 293)**				
**Continuous and count variables**	**Mean** **(SD)**	**Median**	**P25**	**P75**	**Mean** **(SD)**	**Median**	**P25**	**P75**	P-Value*
Duration of follow-up time, months	84.6 (5.9)	84.1	79.9	88.0	85.7 (6.9)	85	79.9	88.7	0.54
Number of references cited	29.3 (13.1)	28.0	20.0	36.0	27.7 (12.5)	25.0	19.0	34.0	0.58
Page length	16.9 (7.2)	16.0	11.0	20.0	17.0 (8.0)	15.0	11.0	20.0	0.28
Number of authors	2.6 (1.1)	2.0	2.0	3.0	2.4 (1.0)	2.0	2.0	3.0	0.42
SCImago Journal Rank indicator (2010)	2.0 (1.2)	1.9	1.1	2.1	2.0 (1.4)	1.4	1.1	2.3	0.71
Journal h-index	61.0 (29.3)	55.0	44.0	70.0	60.3 (31.0)	51.0	36.0	72.0	0.81
Number of publications by the most published author	139.9 (126.0)	107.0	53.0	174.0	136.7 (170.8)	89.0	47.0	168.0	0.80
Total number of publications among authors	213.4 (196.2)	151.0	78.0	262.0	192.9 (210.8)	127.0	62.0	256.0	0.92

* P-values were obtained from generalized linear mixed models to account for clustering within journals, with one exception. In the model for “multiple countries represented by authors”, the final covariance matrix was not positive definite due to small cell counts in certain journals; any other technique to account for clustering (e.g. with generalized estimating equations) would result in an even larger p-value.

The bivariate relationships for each abstracted data element with the number of citations (adjusted for length of follow-up time) are presented in Table 2. Articles with code available had somewhat higher citations, on average, than articles without code, but this bivariate association was not statistically significant (rate ratio = 1.23, 95% CI = 0.96 to 1.57). Articles that were freely accessible on the journal website had about 1.5 times as many citations as articles that were not free (rate ratio = 1.49, 95% CI = 1.05 to 2.11), and articles that were freely accessible in some form on any website had about 1.6 times as many citations as articles that were not free (rate ratio = 1.55, 95% CI = 1.23 to 1.96). Most of the continuous and count-level variables were also associated with the number of citations, including higher numbers of references cited, longer articles, articles with more authors, higher SCImago Journal Rank indicators, and higher journal h-indices.

**Table 2:** Bivariate associations between citation counts and article, author, and journal characteristics.

Characteristics that are categorical variables	Number of citations				
	N	Mean	SD	Rate Ratio^†^	95% CI^†^
Code available					
No	293	14.7	35.8	Reference	
Yes	135	21.1	44.7	1.23	(0.96, 1.57)
At least 1 author is from a US Institution					
No	217	16.3	47.6	Reference	
Yes	211	17.1	27.2	1.05	(0.84, 1.31)
Multiple countries represented by authors					
No	317	14.9	24.9	Reference	
Yes	111	21.7	63.7	1.06	(0.83, 1.35)
Multiple institutions represented by authors					
No	175	12.9	19.3	Reference	
Yes	253	19.3	47.8	1.11	(0.89, 1.38)
Evidence that at least 1 author is not a methodologist					
No	404	16.8	39.8	Reference	
Yes	24	14.0	17.3	1.22	(0.77, 1.93)
Types of examples presented in paper					
No example provided	18	12.4	10.9	Reference	
Simulation only	81	12.0	18.9	0.89	(0.50, 1.59)
Real-life example	329	18.1	43.2	1.08	(0.63, 1.86)
Manuscript deposited into PubMed Central					
No	359	16.8	41.4	Reference	
Yes	69	16.2	22.2	1.29	(0.95, 1.75)
Manuscript freely accessible on journal website					
No	307	13.1	19.0	Reference	
Yes	121	25.7	65.9	1.49	(1.05, 2.11)
Manuscript freely accessible on any website (n, % yes)					
No	156	9.0	10.3	Reference	
Yes	272	21.1	47.6	1.55	(1.23, 1.96)
**Characteristics that are continuous or count variables**		Correlation*	**Rate Ratio**^†^		**95% CI**^†^
Number of references cited ^††^		0.31	1.28		(1.17, 1.39)
Page length^††^		0.17	1.52		(1.29, 1.79)
Number of authors^††^		0.06	1.12		(1.01, 1.25)
SCImago Journal Rank indicator (2010) ^††^		0.26	1.24		(1.13, 1.36)
Journal h-index^††^		0.24	1.07		(1.02, 1.13)
Number of publications by the most published author^††^		0.14	1.01		(1.00, 1.01)
Total number of publications among authors^††^		0.16	1.01		(1.00, 1.01)
Duration of follow-up time (months) ^††^		-0.04	1.03		(0.85, 1.26)

CI: Confidence Interval

^**†**^ Rate ratios and 95% CI’s were obtained using generalized linear mixed models.

^**††**^ For **most** continuous and count variables, the rate ratios reflect the fold-change in number of citations associated with a **10-unit increase** in the independent variable. Exceptions are for SCImago Journal Rank indicator (2010) and Number of authors, for which the rate ratios reflect a **1-unit increase**.

* Spearman correlation with number of citations

The final multivariable generalized linear mixed model is summarized in Table 3, and a number of interesting relationships were noted. All of the reported rate ratios, 95% confidence intervals, and p-values are adjusted for all factors included in the model. A key interaction was identified between having code available and whether the article was freely accessible on the journal website. For articles that were not freely accessible on the journal’s website, having code available appeared to offer no boost to the number of citations (rate ratio = 0.96, 95% CI = 0.74 to 1.24, p = 0.74); however, for articles that were freely accessible on the journal’s website, having code available was associated with a 2-fold increase in the number of citations (rate ratio = 2.01, 95% CI = 1.30 to 3.10, p = 0.002). Having the articles freely accessible on any website increased the citation counts by another 50% (rate ratio = 1.47, 95% CI = 1.15 to 1.88, p = 0.002). Each additional 10 references cited was associated with a 19% increase in the number of citations (rate ratio = 1.19, 95% CI = 1.09 to 1.30, p = 0.0001). Although page length varied considerably (ranging from 1 to 54 pages), longer articles were cited more often than shorter articles; for example, articles that were 20 pages long had about 1.3 times as many citations, on average, compared with articles that were 10 pages long. The strongest association (i.e. the association with the smallest p-value) was noted for the 2010 SCImago Journal Rank indicator, which exhibited a 21% increase in citations for a 1-unit increase in the SJR indicator (rate ratio = 1.21; 95% CI = 1.11 to 1.32). A small but significant association was also noted between citation counts and total number of publications among authors (i.e. a 1% increase in citation counts with every 10 additional publications). All significant associations would remain significant at a Benjamini-Hochberg [21] false discovery rate of 5%, with the exception of the association between citation counts and total number of publications among authors. When the sensitivity analyses were conducted to determine whether our findings were similar when the natural logarithm of the number of citations plus 1 was treated as the dependent variable in the multivariable model or when several articles with extremely high number of citations were excluded from the multivariable analyses, the study findings remained essentially unchanged. The interaction between having code available and whether the article was freely available on the journal website remained highly significant in each case, and all other previously identified statistically significant associations remained significant.

**Table 3:** Final multivariable model predicting citation count, using a generalized linear mixed model with random journal effects included.

Article characteristic	Regression Coefficient Estimate	Regression Coefficient Std Err	Rate Ratio^†^	95% CI^†^	P-value
At least 1 author is from a US Institution	-0.057	0.111	0.94	(0.76, 1.18)	0.61
Multiple countries represented by authors	0.001	0.135	1.00	(0.77, 1.31)	0.99
Multiple institutions represented by authors	-0.007	0.125	0.99	(0.78, 1.27)	0.96
Evidence that at least 1 author is not a methodologist	0.063	0.229	1.07	(0.68, 1.67)	0.78
Types of example presented in paper					
No example provided	0	-	Reference	-	
Simulation only	0.048	0.280	1.05	(0.60, 1.82)	0.86
Real-life example	0.048	0.261	1.05	(0.63, 1.75)	0.85
Manuscript submitted to PubMed Central	NI				
Manuscript freely accessible on any website	0.386	0.124	1.47	(1.15, 1.88)	0.002
Number of references cited ^††^	0.017	0.004	1.19	(1.09, 1.30)	0.0001
Page length^††^	0.024	0.008	1.27	(1.08, 1.49)	0.004
Number of authors	NI				
SCImago Journal Rank indicator (2010) ^††^	0.189	0.044	1.21	(1.11, 1.32)	<0.0001
Journal h-index	NI				
Number of publications by the most published author	NI				
Total number of publications among authors^††^	0.0005	0.0003	1.01	(1.00, 1.01)	0.04
Duration of follow-up time (months) ^††^	0.007	0.009	1.08	(0.91, 1.28)	0.39
Manuscript freely accessible on journal website x Code available					
Effect of code for articles not freely available	-0.043	0.132	0.96	(0.74, 1.24)	0.74
Effect of code for articles that are freely available	0.698	0.221	2.01	(1.30, 3.10)	0.002

Std Err: Standard Error; CI: Confidence Interval; NI: not included in multivariable model due to multicollinearity

^**†**^ Rate ratios and 95% CI’s were obtained using a generalized linear mixed model.

^**††**^ For continuous and count variables, the rate ratios reflect the fold-change in number of citations associated with a **10-unit increase** in the independent variable, with one exception; for SCImago Journal Rank indicator (2010), the rate ratio reflects a **1-unit increase**.

## Discussion

In this study characterizing 428 articles summarizing the development of new biostatistics methods, we found a number of article, author, and journal characteristics that were associated with increased citation counts over about seven years of follow-up. These significant bivariate relationships included the article being freely available, having computer programming code available, higher number of references, longer page length, higher numbers of authors, higher SCImago Journal Rank indicator, higher journal h-index, and higher total numbers of publications among the article’s authors. In a multivariable model, having computer code made available was associated with a 2-fold increase in citation counts over the 7-year time period for articles freely available on the journal website but not for articles that were not freely available. A number of other associations with citation counts remained statistically significant in the multivariable model, with the strongest impact on citation rates coming from SJR, which showed a 21% increase in citations for every 1-unit increase in the SJR indicator.

While some of these associations (i.e., number of references and SJR indicator) are particularly strong even after controlling for other factors, it is possible that unmeasured confounders could explain these associations. Although we observed higher citation rates when computer programming code was provided for articles freely available on journal websites, we cannot be certain that merely providing code for one’s new methods will enhance future citations. Similarly, it may be that higher quality articles simply tend to have higher numbers of references, more pages, more authors, and more publications among its authors; thus, we do not suggest that intentionally trying to increase these factors would directly translate to higher numbers of citations. Factors that could potentially confound the observed associations could include variables that would be extremely difficult to capture such as number of formal/informal presentations of the new method, whether the authors work at institutions that are better at supporting translation of scientific findings, or other less tangible factors such as notoriety/popularity of the authors, how socially connected the authors are to potential users of their methods, quality of the article, or relevance of the topic to other investigators/disciplines. Identifying the impact of such confounders or latent variables is a topic for future research.

Barriers to the adoption of new biostatistics methods by other ‘users’ have been described in the literature to some extent. Pullenayegum et al. (2016) note the following barriers: lack of expertise in the area, lack of software, and lack of time needed to understand and utilize new methods. Providing computer code to the reader may be one means of enhancing adoption of new methods [22] or at a minimum assisting in the reproducibility process. In this review, we observed a variety of different types of computer code being provided, with R scripts and packages being the most common. Although we did not investigate reasons why methodologists do not make code available, perhaps biostatisticians, like many scientists, feel a burden to have multiple publications within a short time frame for promotion and may thus forego the added steps necessary to provide code to the reader [23].

Our study has several strengths and limitations worth noting. We included a broad range of journals in which new biostatistical methods are summarized, and the sample size was sufficiently large to allow us to investigate a number of potential associations with citation frequencies. While we had sufficient power for our primary question of interest (relationship with providing software code), it should be noted that we did not have sufficient power to assess all other covariates included in the multivariable model. For example, 77% of the articles provided a “real-life example”, rendering insufficient power to detect a significant association for this covariate. Although there was some subjectivity in the article assessments, relevant characteristics were abstracted by two of three biostatisticians working independently. Because there are so many journals that highlight new biostatistical methods, we could not include them all in this bibliometric analysis; however, we did include all mentioned in responses to in an informal e-mail survey of biostatisticians. Our study focused on papers published in 2010, and it may be that authors have already started providing software code on a more consistent basis; one journal has even recently published guidelines on structuring code to assist authors [13], and many now require or strongly recommend that data and code be submitted with the manuscript [11, 24–28]. Such guidelines will help ensure that new methods are readily available for use by others. By focusing only on 2010 publications, however, we minimized substantial differences in follow-up time and were thus able to look forward for 7 years (until 2017) to capture citation counts. We also recognize that the sciences of biostatistics and bioinformatics are often blurred and that we did not specifically address new bioinformatics methods; whether our findings hold true in related fields is an area of future research. Finally, there are ways in which authors of new methods can ‘market’ their findings and encourage knowledge transfer that are beyond what can be captured in a study such as this one; aspects such as authors’ involvement in professional societies, the degree to which they speak in front of captive audiences, and personal web pages were beyond the scope of this study.

These analyses shed new insight into factors associated with citation rates of biostatistical methods articles. We have demonstrated an association between citation rates and several article, journal, and author characteristics, including whether programming code is available, whether the manuscript is freely accessible, greater numbers of references, and length of article. For biostatisticians publishing their novel methods, ensuring that relevant computer code is available to readers and that their articles are freely available to readers are goals worth striving for, given their relationship with other investigators’ use of the new methods.

## Supporting information

S1 Checklist PRISMA checklist. (DOC)

S1 Dataset Dataset used to generate Tables 1, 2 and 3. (XLS)

S1 File SAS program to generate information displayed in Tables 1, 2 and 3. (SAS)
